# Experiences of sexual violence and their associations with suicidal ideation and behavior in the past year: an analysis of adults from the 2021 Ace Community Survey

**DOI:** 10.5249/jivr.v15i2.1843

**Published:** 2023-07

**Authors:** Brian N. Fink

**Affiliations:** ^*a*^ University of Toledo Health Science Campus, Department of Population Health, 3000 Arlington Ave., MS# 1027, Toledo, Ohio 43614, United States of America.

**Keywords:** Sexual violence, Suicidal ideation, Asexual identity

## Abstract

**Background:**

This research assessed forms of sexual violence and their associations with suicidal ideation among adults identifying on the asexual identity spectrum.

**Methods:**

A secondary data analysis was conducted among adults, identifying on the asexual spectrum of asexual, gray-asexual, or demisexual, from the 2021 Ace Community Survey (n = 8,715). Multiple logistic regression analyses determined potential associations between sexual violence and suicidal ideation, adjusting for the covariates of age group, gender, education, racial/ethnic minority, employment, and asexual spectrum identity.

**Results:**

Demisexual individuals were at statistically greater odds of suicidality compared to gray-asexual and asexual individuals. Sexual violence victims were more likely to be suicidal compared to non-victims. This was especially true for attempted rape and suicidal consideration (OR = 2.10, 95% CI (1.60, 2.75), planning (OR = 1.76, 95% CI (1.32, 2.34), and attempts (OR = 3.15, 95% CI (2.07, 4.81).

**Conclusions:**

Asexual victims of sexual violence were more likely to be suicidal compared to non-victims. Demisexual individuals were more likely to be suicidal compared to asexual individuals. These findings demonstrate the need for additional research on sexual violence and suicide.

## Introduction

A sexuality may be defined as lacking sexual attraction or experiencing no sexual feelings or desires.^1-3^ The prevalence of asexual individuals is not certain, but prior studies have indicated this may be between less than one percent.^4,5^ However, within these estimates, the specific distribution of asexual individuals identifying as asexual, gray-asexual, or demisexual, is uncertain. Demisexual is defined as someone who experiences sexual attraction after developing a strong emotional connection with another person. Gray-asexual is defined as someone who can experience or has experienced sexual attraction, infrequently, but expects their relationships not to involve sexual attraction.^2^

Asexual individuals are a sexual and gender minority (SGM) population that may be at an increased risk of recognizing sexual aggression as violence.^6^ Though violence occurs in these populations, research is limited regarding different experiences of sexual violence and their potential association with suicidal ideation.^7^ This is particularly troubling because the short- and long-term health effects resulting from sexual violence can adversely affect and possibly end life. 

As part of the minority stress theory, suicidality may be attributed to stressors such as violence, victimization, and discrimination, particularly among those within the lesbian, gay, bisexual, transgender, queer, intersex, asexual (LGBTQIA+) community.^8^ Asexual individuals may be discriminated due to the erasure of asexuality as an orientation^9-10^ and structurally due to the lack of legal benefits and privileges for those involved in conjugal partnerships.^11-12^ Victimization and discrimination against asexual individuals may lead them to suicidal ideation and self-harm.^13-15^ In fact, suicide may be a response to minority stress and asexual individuals may view suicide as a method to no longer deal with victimization and discrimination.^16-17^

Among the general population, sexual violence is associated with suicidal behaviors.^18^ This includes sexual assault survivors having a five-fold increase in past-year suicide attempts and a higher prevalence of lifetime and past-year suicidal ideation compared to those who have never been assaulted.^19-20^ Suicidal thoughts and suicide attempts in the past year have been found to be more common among LGBTQ+ individuals who experience verbal abuse, physical abuse, or violence.^7^ While this suggests that sexual orientation is associated with suicidal ideation, it is unclear if sexual violence is associated with suicidal ideation among asexual individuals.

However, this study combined all participants identifying on the asexual spectrum as “asexual” and included children – to which some institutional review boards (IRB) will not approve, even for research considered to be “non-human subjects.” 

Thus far, there is limited published research studying any form of violence and possible associations with suicidality in the asexual population. Researchers studying the 2011 Chicago Youth Risk Behavior Survey found sexual minority youth are at an increased risk of suicidality due to victimization from being threatened or injured with a weapon and experiencing harassment.^21^ A study using the 2020 Ace Community Survey indicated that victims of violence were more likely to consider, plan, and attempt suicide than non-victims.^22^ In this study, both children and adults were included in the sample and nearly one-third of asexual individuals reported suicidal ideation, 10.6% had planned suicide, and 2.7% had attempted suicide in the past year.^22^ However, this study combined all participants identifying on the asexual spectrum as “asexual” and included children – to which some institutional review boards (IRB) will not approve, even for research considered to be “non-human subjects.”

As stated previously, there are differences, particularly related to sexual attraction, among those who identify as asexual, demisexual, and gray-asexual. Therefore, an objective of this research is to investigate how sexual violence, as well as other characteristics, may be associated with suicidal consideration, planning, and attempts among each of these asexual spectrum identities. The two hypotheses of this research are the following: 1) Adult sexual violence survivors who identify on the asexual spectrum will be more likely to consider, plan, and attempt suicide in the past twelve months compared to those who have not experienced sexual violence; and 2) Those who identify as demisexual or gray-asexual may be more likely to consider, plan, and attempt suicide compared to those who identify as asexual. As asexuality is about sexual attraction,^2^ those who identify as demisexual or gray-asexual, are more likely to experience sexual attraction than those who identify as asexual. Thus, they may have a greater probability to be involved in a situation that may escalate to sexual violence, which could increase their likelihood of suicidal ideation in the past twelve months.

## Methods 

***Participants and Procedures***

The appropriate ethical approval was received from the University of Toledo Biomedical Institutional Review Board (IRB) to conduct this secondary data analysis of the 2021 Ace Community Survey. This study was approved within the category of non-human subjects research. The Ace Community Survey is a survey conducted each year, online, of asexual communities, run by the Ace Community Survey Team. The survey follows the demographics and the well-being of asexual communities to provide a communal needs assessment.^23^ Targeted and snowball sampling were used to recruit participants, sharing the survey link to asexual websites such as the Asexual Visibility Education Network (AVEN) and The Asexual Agenda. The survey was also distributed through asexual-themed groups on various social media platforms including, but not limited to, Facebook, Instagram, and Twitter. Those who were interested in could access and complete the online survey that was created in Google Forms.

***Dependent Variable***

The outcome of interest was suicidal ideation, which included considering, planning, and attempting suicide. Suicidal ideation was assessed through the following questions from the 2021 Ace Community Survey: “During the past twelve months, did you ever seriously consider suicide; did you make a plan about how you would attempt suicide; and how many times did you actually attempt suicide?”^24^ If a participant indicated at least one attempted suicide, they were considered to have ever attempted suicide in the past year. Those indicating zero attempts were considered to not have attempted suicide in the past year.

***Independent Variables***

***Sexual Violence***

The survey included ten questions inquiring about various types of sexual violence, each allowing the respondent to answer how many times they had experienced each type of violence (Table 1).^24^ These violence types included verbal sexual harassment in a public setting, unwanted sexual contact, sexual coercion, alcohol/drug-facilitated completed rape, attempted rape by physical force, and completed rape by physical force. If respondents answered they had been a victim of a type of violence at least once, they were classified as a violence victim for that type. For those who responded with “zero times,” they were classified as non-victims.

**Table 1:** Definitions of Sexual Violence Types

**Verbal Sexual Harassment in Public Setting **
In the last twelve months, how many times has someone sexually harassed you while you were in a public place in a way that made you feel unsafe?^1^
**Unwanted Sexual Contact **
In the last twelve months, how many times has someone attempted to or kissed you in asexual way, fondled, groped, grabbed or touched you without your consent and/or in a way that made you feel unsafe? ^1^
In the last twelve months, how many times has someone exposed their sexual body parts, made you show your own sexual body parts, or made you look at sexual photos or videos when you didn't want it to happen? ^1^
**Sexual Coercion **
How many people have ever had vaginal, anal, oral, or manual sex with you, or put fingers or an object into your vagina, mouth, or anus in the following circumstances:
After they pressured you by telling you lies, or making promises about the future that they knew were untrue? ^1^
How many people have ever had vaginal, anal, oral, or manual sex with you, or put fingers or an object into your vagina, mouth, or anus in the following circumstances:
After they pressured you by threatening to end your relationship or threatening to spread rumors about you? ^1^
How many people have ever had vaginal, anal, oral, or manual sex with you, or put fingers or an object into your vagina, mouth, or anus in the following circumstances:
After they pressured you by wearing you down by repeatedly asking for sex or showing they were unhappy?^1^
How many people have ever had vaginal, anal, oral, or manual sex with you, or put fingers or an object into your vagina, mouth, or anus in the following circumstances:
After they pressured you by using their influence or authority over you, for example a boss or a teacher? ^1^
**Alcohol/Drug-Facilitated Rape, Completed **
How many people have ever had vaginal, anal, oral, or manual sex with you, or put fingers or an object into your vagina, mouth, or anus in the following circumstances: When you were drunk, high, or passed out and unable to consent? ^1^
**Physically Forced Rape, Attempted **
In the last twelve months, how many times has someone threatened you with any kind of penetrative sexual act (whether with them-selves or on your own) without your consent, but it *did not* happen?^1^
**Physically Forced Rape, Completed **
How many people have ever had vaginal, anal, oral, or manual sex with you, or put fingers or an object into your vagina, mouth, or anus in the following circumstances:
Using force or threats to physically harm you? ^1^

1. Questions from the Sexual Violence section of the 2021 Ace Community Survey

The National Intimate Partner and Sexual Violence Survey (NISVS) defines “rape” as “any completed or attempted unwanted vaginal (for women), oral, or anal penetration through the use of physical force (such as being pinned or held down, or by the use of violence) or threats to physically harm and includes times when the victim was drunk, high, drugged, or passed out and unable to consent.”^25^

The NISVS defines “ sexual coercion” as “unwanted sexual penetration that occurs after a person is pressured in a non-physical way”, including “unwanted vaginal, oral, or anal sex after being pressured in ways that included being worn down by someone who repeatedly asked for sex or showed they were unhappy; feeling pressured by being lied to, being told promises that were untrue, having someone threaten to end a relationship or spread rumors; and sexual pressure due to someone using their influence or authority.” ^25^

The NISVS defines “unwanted sexual contact” as “unwanted sexual experiences involving touch but not sexual penetration, such as being kissed in a sexual way, or having sexual body parts fondled, groped, or grabbed.” ^25^

***Asexual Identity***

Asexual identity was determined from the following, “Which of the following labels do you most closely identify with?” followed by the options of “asexual, gray-asexual, demisexual, and questioning if asexual/gray-asexual/demisexual.”^23^

***Gender Identity***

This was determined from the following, “Which of the following best describes your gender identity? (check all that apply)”, followed by the options of “man or male, woman or female, non-binary, agender, androgynous, bigender, demigirl, demiguy, genderfluid, genderqueer, neutrois, no gender, questioning or unsure, and other”.^24^ Due to the “check all that apply” option, there were hundreds of combinations of gender identities. For analysis simplification, these individuals were classified as “non-binary.” All participants in this study did include those who also identified as transgender. 

***Data Analysis***

The data analysis program utilized was Statistical Package for the Social Sciences (SPSS) version 29.0 (Armonk, NY: IBM Corp.). Demographic characteristics, as well as sexual violence events and occurrences of suicidal ideation were determined (Table 2). Since the variables for sexual violence and suicide are nominal, a correlation matrix using phi from cross-tabulated chi-square analyses, (Table 3) displays the bivariate correlations between each type of sexual violence and suicidal ideation. Logistic regression was used to model the effects of these demographics, as well as sexual violence, on suicidal consideration, planning, and attempts (Table 4).

**Table 2:** Demographic Characteristics, Sexual Violence Experiences, and Suicidality of Participants (n = 8,715).

Demographic Characteristics	N (%)/Mean (SD)		N (%)
**Age**	25.1 (7.28)	**Asexual Identity**	
		Asexual	5,821 (66.8%)
**Gender**		Gray-asexual	975 (11.2%)
Woman or female	3,793 (43.5%)	Demisexual	1,070 (12.3%)
Man or male	862 (9.9%)	Questioning	849 (9.7%)
Non-binary	4,060 (46.6%)		
Racial/Ethnic Minority		Sexual Violence Types Unwanted Contact	
Yes	941 (10.8%)	Verbal harassment	2,043 (23.4%)
		Non-consent grope	1,124 (12.9%)
**Education**		Exposed or look	832 (9.5%)
Less than Secondary	331 (3.5%)		
Secondary Education	1,555 (16.7%)	**Coercion**	
Some College	3,395 (36.4%)	Coercion lies/promises	878 (10.1%)
Associate’s	480 (5.1%)	Coercion threats	433 (5.0%)
Bachelor’s	2,249 (24.1%)	Coercion wear down	1,575 (18.1%)
Master’s	1,053 (11.3%)	Coercion authority	225 (2.6%)
Professional	103 (1.1%)		
Doctoral	114 (1.2%)	**Rape attempted or completed**	
Missing	44 (0.5%)	Alcohol/drug completed	807 (9.3%)
		Physical rape attempted	298 (3.4%)
**Employment Status**		Physical rape completed	433 (5.0%)
Employed, paid, part/full	4,430 (50.8%)		
Not employed and looking and/or on disability	956 (11.0%)	**Suicide**	
Not employed and not looking	392 (4.5%)	Suicidal ideation	1,993 (22.9%)
Student	2,707 (31.1%)	Suicide planning	1,266 (14.5%)
Caregiver/Parent	51 (0.6%)	Suicide attempt(s)	249 (2.9%)
Retired	23 (0.3%)		
Volunteer	103 (1.2%)		
Missing	53 (0.6%)		

**Table 3:** Correlation matrix of sexual violence and suicidality in the past twelve months.

	Consider Suicide ϕ (p)	Plan Suicide ϕ (p)	Attempt Suicide ϕ (p)
Verbal harassment in public setting	0.152 (<.01)	0.136 (<.01)	0.079(<.01)
Attempted or kissed in sexual way, fondled, groped, grabbed	0.169 (<.01)	0.130 (<.01)	0.113 (<.01)
Exposed body parts, look at photos or videos	0.143 (<.01)	0.151 (<.01)	0.120 (<.01)
Lies, or making promises about the future that they knew were un-true?	0.100 (<.01)	0.102 (<.01)	0.075 (<.01)
Threatening to end your relation-ship or threatening to spread rumors about you?	0.092 (<.01)	0.085 (<.01)	0.069 (<.01)
Wearing you down by repeated-ly asking for sex or showing they were unhappy?	0.078 (<.01)	0.066 (<.01)	0.059 (<.01)
Using their influence or authority over you, for example a boss or a teacher?	0.087 (<.01)	0.064 (<.01)	0.095 (<.01)
Alcohol/Drug-Related Completed Rape	0.087 (<.01)	0.075 (<.01)	0.069 (<.01)
Physically-forced Rape, Attempt-ed	0.156 (<.01)	0.136 (<.01)	0.173 (<.01)
Physically-forced Rape, Complet-ed	0.097 (<.01)	0.090 (<.01)	0.113 (<.01)

**Table 4:** Adjusted logistic regression modeling the effects of demographics and sexual violence on suicidality in the past twelve months.

	Suicidal Ideation		Suicidal Planning		Suicide Attempts	
	AORa 95% CIb	p	AORa 95% CIb	p	AORa 95% CIb	p
**Age **	0.97 (0.96,0.98)	<.001	0.97 (0.96,0.99)	<.001	0.94 (0.91,0.97)	<.001
**Gender**						
Woman or female	1 [Reference]		1 [Reference]		1 [Reference]	
Man or male	1.58 (1.29,1.94)	<.001	1.63 (1.28,2.09)	<.001	1.59 (0.94,2.69)	.08
Non-binary	1.56 (1.38,1.77)	<.001	1.61 (1.39,1.87)	<.001	1.57 (1.14,2.17)	.01
**Race/Ethnic Minority**						
No	1 [Reference]		1 [Reference]		1 [Reference]	
Yes	1.23 (1.03,1.48)	.02	1.15 (0.93,1.42)	.21	1.51 (1.02,2.23)	.21
Unsure	1.00 (0.77,1.29	.99	0.91 (0.66,1.24)	.54	0.61(0.28,1.33)	.04
**Asexual Identity**						
Asexual	1 [Reference]		1 [Reference]		1 [Reference]	
Gray-asexual	1.07 (0.90,1.29)	.44	1.07 (0.86,1.32)	.55	1.04 (0.66,1.64)	.87
Demisexual	1.28 (1.08,1.52)	<.001	1.10 (0.89,1.35)	.38	1.51 (1.02,2.23)	.04
Questioning	0.95 (0.77,1.16)	.61	1.00 (0.79,1.27)	.98	0.91 (0.54,1.53)	.71
**Education **						
Less than Secondary	1 [Reference]		1 [Reference]		1 [Reference]	
Secondary Education	0.81 (0.58,1.13)	.21	0.84 (0.57,1.23)	.36	0.46 (0.25,0.83)	.01
Some College	0.72 (0.52,0.99)	.04	0.75 (0.52,1.08)	.12	0.46 (0.26,0.82)	.01
Associate’s	0.62 (0.41,0.92)	.02	0.68 (0.43,1.08)	.10	0.51 (0.23,1.11)	.09
Bachelor’s	0.53 (0.38,0.75)	<.001	0.48 (0.32,0.71)	<.001	0.26 (0.13,0.51)	<.001
Master’s	0.42 (0.29,0.62)	<.001	0.50 (0.32,0.78)	<.001	0.24 (0.10,0.58)	.001
	Suicidal Ideation		Suicidal Planning		Suicide Attempts	
	AORa 95% CIb	p	AORa 95% CIb	p	AORa 95% CIb	p
Professional	0.56 (0.28,1.13)	.11	0.75 (0.34,1.64)	.47	N/A	N/A
Doctoral	0.36 (0.18,0.75)	.01	0.47 (0.21,1.09)	.08	N/A	N/A
**Employment**						
Employed	1 [Reference]		1 [Reference]		1 [Reference]	
Unemployed, looking/disability	1.94 (1.62,2.33)	<.001	1.92 (1.56,2.35)	<.001	2.27 (1.53,3.37)	<.001
Unemployed and not looking	1.28 (0.97,1.70)	.07	1.09 (0.78,1.53)	.62	0.76 (0.34,1.69)	.50
Full-time student	0.88 (0.76,1.02)	.09	0.99 (0.83,1.17)	.87	0.95 (0.67,1.36)	.79
Parent/Caregiver	0.83 (0.38,1.81)	.63	0.40 (0.12,1.37)	.15	N/A	N/A
Retired	1.27 (0.26,6.03)	.77	1.02 (0.13,8.14)	.99	N/A	N/A
Volunteer	0.74 (0.42,1.28)	.28	0.66 (0.33,1.32)	.24	0.67 (0.16,2.89)	.60
**Unwanted Contact**						
Verbal harassment	1.60 (1.40,1.83)	<.001	1.69 (1.45,1.98)	<.001	1.41 (1.02,1.95)	.04
Non-consent grope	1.67 (1.42,1.96)	<.001	1.37 (1.14,1.64)	<.001	1.65 (1.16,2.34)	.001
Exposed or look	1.51 (1.27,1.80)	<.001	1.86 (1.53,2.24)	<.001	1.75 (1.24,2.48)	.01
**Rape attempted or completed**						
Alcohol/drug completed	1.51 (1.24,1.83)	<.001	1.41 (1.13,1.76)	.10	1.46 (0.95,2.24)	.08
Physical rape attempted	2.10 (1.60,2.75)	<.001	1.76 (1.32,2.34)	<.001	3.15 (2.07,4.81)	<.001
Physical rape completed	1.44 (1.13,1.84)	.01	1.42 (1.08,1.86)	.01	1.61 (1.01,2.58)	.04

aAOR, adjusted odds ratio bCI, confidence interval

## Results

There were 8,715 participants identifying on the asexual spectrum, representing 54 different countries, included in this study. A total of 3,793 (43.5%) identified as female (80 of whom were transgender) and 862 (9.9%) whom identified as male (126 of whom were transgender), and 4,060 (46.6%) whom identified as non-binary (1,200 of whom were transgender) (Table 2). Most of the participants identified as asexual (66.8%), followed by demisexual (12.3%), gray-asexual (11.2%), and questioning (9.7%). There were 609 adults (6.5%) who did not answer the asexual identity question and were therefore excluded from the analysis. Age ranged from 18 to 85 years with a mean age of 25.1 years and a standard deviation of 7.3 years. 

Various forms of sexual coercion in the past year were reported among asexual spectrum individuals, ranging from 2.6% to 18.1% victimization (Table 2). Unwanted sexual contact was reported by 9.5% (exposed to body parts or forced to look at photos) to 12.9% (grabbed, groped, touched without consent), while verbal sexual harassment was reported by 23.4% of this study population (Table 2). Attempted rape in the past year (3.4%), completed rape by physical force (5.0%), and completed rape using alcohol or other drugs (9.3%) was also reported. More than one-in-five (22.9%) reported suicidal consideration, nearly one-in-six (14.5%) reported suicide planning, and 2.9% reported attempting suicide in the past twelve months (Table 2).

Cross-tabulation analyses using the phi coefficient (ϕ) were conducted to measure the strength of association between the nominal variables of sexual violence and suicidal ideation (Table 3). While all values were statistically significant and indicated positive associations between sexual violence and suicide, they were relatively weak, as the variable with the greatest value was attempted rape and suicidal attempt (ϕ = 0.173, p <.01).

The multiple logistic regression models excluded the sexual coercion variables from the model in SPSS due to multicollinearity and, therefore, not contributing to the model. However, the remaining sexual violence variables regarding sexual harassment, unwanted sexual contact, and rape were all included and each of these variables had an increased, adjusted odds of suicidal ideation, planning, and attempts that were statistically significant or borderline statistically significant (Table 4). For example, those asexual individuals who were victims of attempted rape in the past year, had a greater odds of suicidal ideation (OR = 2.10, 95% CI (1.60, 2.75), suicidal planning (OR =1.76, 95% CI (1.32, 2.34), and suicidal attempts (OR = 3.15, 95% CI (2.07, 4.81) in the past year. Similarly, those who were victims of verbal sexual harassment had a greater odds of suicidal ideation (OR = 1.60, 95% CI (1.40, 1.83), suicidal planning (OR =1.69, 95% CI (1.45, 1.89), and suicidal attempts in the past twelve months (OR = 1.41, 95% CI (1.02, 1.95).

Demisexual and gray-asexual individuals had a slightly greater adjusted odds of suicidal ideation, planning, and attempts in the past year, compared to asexual individuals (Table 4). Statistically significant associations were observed for demisexual individuals and suicidal ideation (OR = 1.28, 95% confidence interval (CI) (1.08, 1.52)) and suicide attempts in the past twelve months (OR = 1.51, 95% CI (1.02, 2.23) Table 4). 

With increased education, there was a general decline in the odds of suicidal ideation, planning, and attempts in the past year. Males and non-binary individuals were more likely to consider, plan, and attempt suicide, compared to females (Table 4). Those asexual individuals who reported as racial/ethnic minorities were at statistically greater odds of both suicidal ideation and suicidal attempts in the past twelve months (Table 4). Those who were unemployed but looking for a job had greater odds of considering, planning, and attempting suicide in the past twelve months than not only those who were employed, but also those who were unemployed and not looking for a job (Table 4).

## Discussion

This study, with a sample of 8,715 individuals from around the globe who identify on the asexual spectrum, provides insight into the potential associations of sexual violence and suicidality. All forms of sexual violence, particularly attempted rape in the past twelve months, was associated with greater odds of suicidal thoughts, planning, and attempts in the past twelve months. These findings are consistent with previous studies that have demonstrated associations between sexual violence and suicide.^8,13-15,17,22,26^

Being forced to look at sexual videos or photos was associated with greater odds of all forms of suicidality among adults. Similar results were obtained from a study of sexual minority youth, where threats to post sexually explicit media without consent were associated with greater odds of suicidal ideation, planning, attempts, and self-harm.^26^ Demisexual individuals had greater odds of suicidal consideration and attempts in the past year compared to those identifying as asexual. Along these lines, a previous study of the 2019 Ace Community Survey demonstrated that individuals identifying as asexual were most likely to score the lowest on measures of sex drive, and personal disposition towards engaging in sex, compared to both gray-asexual and demisexual individuals.^27^ As demi-sexual and gray-asexual individuals, by definition, are more inclined to feel sexual attraction at times^2^, they may have a greater risk of being in a situation that could result in a form of sexual violence.

In this study, both male and non-binary individuals on the asexual spectrum were at a statistically greater odds of all forms of suicidal ideation (consideration, planning, and attempts) compared to females identifying on the asexual spectrum. Prior research has demonstrated that transgender and gender non-conforming individuals have higher rates of suicide compared to the overall population.^28^

Analysis of the 2021 Ace Community Census revealed that racial and ethnic minorities had greater odds of suicidality in the past twelve months compare to those who were not racial and ethnic minorities and those who were employed, respectively. Racial and ethnic discrimination may be a source of traumatic stress and increase the risk of suicidal ideation and suicide attempts.^29^ While those who were unemployed and looking for work were at greater odds of suicidality compared to those who were employed, the odds of suicidality tended to decrease with increasing levels of educational attainment. These findings coincide with prior research which found that minorities were at greater risk of sexual violence due, in part, to socioeconomic factors including poverty, education, and employment status.^30^ Thus, in addition to being a member of a SGM population, those identifying on the asexual spectrum may have additional characteristics that increase their vulnerability to both sexual violence and suicidality.

***Limitations***

The cross-sectional design of the 2021 Ace Community Survey may prevent the drawing of causal inferences between sexual violence and suicidal ideation, planning, and attempts. Though some of the sexual violence (harassment, unwanted contact, and attempted rape) and suicide questions inquired about the previous twelve months, it is unclear if these sexual violence events occurred before any thoughts or actions regarding suicide. Prospective cohort studies are needed to better assess the temporal relationship between all acts of sexual violence and suicide, accounting for variables related to minority stress. 

Though targeted and snowball sampling on asexual-based websites and other social media platforms is useful for reaching difficult-to-access populations such as those part of the asexual spectrum, it may diminish the generalizability of conclusions from this research to all asexual individuals.

Victims of violence, particularly from a spouse, girlfriend, or boyfriend, may not have considered the event as violent. Thus, obtaining a history of sexual violence may be hindered by the questions covered by the 2021 Ace Community Survey. Although the Ace Community Survey questions appear to be the same as those from the NISVS, it is unknown if the NISVS was used to create the 2021 Ace Community Survey questions. More questions and improved wording regarding when these experiences occurred would be helpful for future research. 

## Conclusion

Though asexual individuals appear to be included more frequently in scientific research, sexual violence and suicidality are only beginning to be studied. This research of asexual individuals from around the world helps to fill this nascent area. The results demonstrate that verbal sexual harassment, unwanted sexual contact, and attempted rape within the past year are positively associated with suicidal consideration, suicidal consideration, and suicide attempts – adjusting for numerous variables. Completed rape by physical means and alcohol/drugs was also positively associated with suicidality. Further, there were differences in suicidal consideration, planning, and attempts by asexual spectrum identity, as demisexual individuals had statistically greater odds of both consideration and attempts compared to both gray-asexual and asexual individuals. These findings contribute to the growing research of those who identify on the asexual spectrum.

Authorship Confirmation/Contribution Statement: I am the sole author of this manuscript. Pursuant to the CRediT taxonomy, I conceptualized, cured the data, analyzed, investigated, used the necessary resources with the appropriate software, and did all the writing (including editing) of this manuscript. 
